# The Whole Transcriptome Sequencing Profile of Serum-Derived Exosomes and Potential Pathophysiology of Age-Related Hearing Loss

**DOI:** 10.3390/diagnostics16020248

**Published:** 2026-01-12

**Authors:** Guijun Yang, Zhongqin Xie, Yu Huang, Jing Ke, Ziyi Tang, Zhiji Chen, Shaojing Kuang, Feixian Li, Huan Luo, Qin Lai, Bo Wang, Juhong Zhang, Wei Yuan

**Affiliations:** 1 Department of Otorhinolaryngology Head and Neck Surgery, Chongqing Medical University, Chongqing 400016, China; y402681589@126.com (G.Y.); 15963546215@163.com (Z.X.); 2 Department of Otorhinolaryngology Head and Neck Surgery, Chongqing General Hospital, Chongqing University, Chongqing 401147, China; kejingyx@163.com (J.K.); czj198847@sina.com (Z.C.); george8848@163.com (S.K.); lixxian163@163.com (F.L.); sqwwwlz@163.com (H.L.); jjdlzq@163.com (Q.L.); 18212591512@163.com (B.W.); 3 Xinqiao Community Health Service Center, Shapingba District, Chongqing 400030, China; huangsu104@163.com; 4 CQMPA Key Laboratory for Development and Evaluation of Innovative Biological Products, Chongqing 401121, China; tangziyi@cqifdc.org.cn

**Keywords:** age-related hearing loss, exosomes, lncRNA transcriptome sequencing, lncRNA, mRNA

## Abstract

**Objectives:** To systematically analyze the expression profiles of long non-coding RNAs (lncRNAs) in serum-derived exosomes from patients with age-related hearing loss (ARHL), and to further identify key regulatory lncRNAs involved in the pathogenesis and progression of ARHL. **Methods:** Peripheral blood samples were collected from patients with ARHL and age-matched normal-hearing controls. Serum was separated and exosomes were extracted. The exosomes were identified by nanoparticle tracking analysis (NTA), transmission electron microscopy (TEM), and Western blot. Subsequently, total RNA was extracted from the purified exosomes for lncRNA transcriptome sequencing. Based on the sequencing results, we identified differentially expressed lncRNAs and mRNAs and conducted multi-dimensional functional analysis, including Gene Ontology (GO), Kyoto Encyclopedia of Genes and Genomes (KEGG), Reactome pathway database (Reactome), and Disease Ontology (DO). Finally, four key mRNAs (*THAP2*, *ZNF225*, *MED12*, and *RNF141*) and four differentially expressed lncRNAs (DE-lncRNAs), namely MSTRG.150961.7, ENSG00000273015, MSTRG.336598.1, and ENSG00000273493, were experimentally verified by quantitative real-time polymerase chain reaction (RT-qPCR) technology. **Results:** Exosomes were successfully isolated from serum and confirmed by particle size, morphological examination, and the expression of exosome-labeled proteins. A total of 2874 DE-lncRNAs were identified, among which 988 were downregulated and 1886 were upregulated. Similarly, 2132 DE-mRNAs were detected, among which 882 were downregulated and 1250 were upregulated. GO analysis revealed significant enrichment in biological processes such as “phospholipid binding”, “phosphatidylinositol binding”, “phosphatase binding”, “phosphatidylinositol bisphosphate binding”, “phosphatidylinositol-4,5-bisphosphate binding”, “phosphatidylinositol-3,5-bisphosphate phosphatase activity”. KEGG is significantly enriched in signaling pathways including “Wnt signaling pathway”, “Hippo signaling pathway”, “Cushing syndrome”, and “Nucleocytoplasmic transport”. The functional annotations of Reactome were significantly enriched in biomolecular pathways including “tRNA processing”, “Cellular response to heat stress”, “Extra-nuclear estrogen signaling”, “Metabolism of non-coding RNA”, and “CTNNB1 T41 mutants aren’t phosphorylated”. DO is significantly enriched in diseases or pathological conditions such as “hepatitis”, “bacterial infectious disease”, “cystic fibrosis”, and “vasculitis”. **Conclusions:** *THAP2*, *ZNF225*, *MED12*, and *RNF141* may serve as potential candidate biomarker for ARHL. Additionally, lncRNA MSTRG.150961.7, lncRNA MSTRG.336598.1, and lncRNA ENSG00000273493 may play significant roles in the pathogenesis of this condition.

## 1. Introduction

Age-related hearing loss (ARHL), also known as presbycusis, is the most common type of sensory impairment in the elderly. This disease is characterized by bilateral, symmetrical, and progressive sensorineural hearing loss, and its prevalence increases significantly with age [1]. Presbycusis not only leads to hearing impairment, but is also closely related to cognitive decline [2], increased risk of dementia [3], communication difficulties, social isolation, and depression [4], placing a substantial health and economic burden on affected individuals, their families, and society at large. With the continuing acceleration of population aging, ARHL has become an urgent public health issue that requires greater attention and effective solutions. It is currently believed that the pathogenesis involves multiple alterations in the cochlea and central auditory pathways [5]; however, the precise molecular mechanisms remain incompletely understood. Therefore, further investigation into the key molecular regulatory mechanisms underlying the development and progression of ARHL is of major scientific and clinical importance.

Exosomes are membranous vesicles encapsulated by a lipid bilayer, containing various proteins, nucleic acids (such as lncRNA, circRNA, and mRNA), and lipids [6,7]. They are released by most cell types and facilitate intercellular communication either through receptor-mediated signaling or by delivering functional molecules to recipient cells [8]. In 2020, Breglio and colleagues demonstrated the presence of hair cell-derived exosomes in the perilymph of patients with various inner ear disorders, including Meniere’s disease, conductive/mixed hearing loss, and genetic sensorineural hearing loss [9]. Exosomes released from the inner ear tissue have a protective effect on aminoglycoside-induced hair cell death [10]. However, at present, there is still very little research on the role of exosomes in the pathogenesis of ARHL.

LncRNAs are a class of RNA molecules that do not encode proteins and lack open reading frames. Previous studies have performed preliminary analyses of lncRNA expression profiles in the cochlea of aged mice based on existing datasets [11]. Mechanistic investigations have revealed that the Arid5b/AW112010 signaling pathway is specifically activated in the cochlea of aged mice, where it promotes mitochondrial biogenesis and helps maintain mitochondrial function [12]. Furthermore, lncRNA H19 has been shown to alleviate oxidative stress damage to HEI-OC1 cochlear hair cells by regulating the miR-653-5p/SIRT1 axis, thereby delaying the progression of ARHL [13]. Our research group successfully constructed the lncRNA transcription profile of the cochlear base turn in aged mice in the previous work and found that lncRNA Mirg upregulates Foxp1 expression and promotes oxidative stress-induced apoptosis of HEI-OC1 cells [14]. However, current research remains largely confined to cellular and animal models. The expression profiles of lncRNAs in serum exosomes from patients with ARHL have not yet been reported, highlighting an urgent need for further validation in human populations and functional exploration.

CircRNAs are a class of non-coding RNA molecules that have received extensive attention in recent years [15,16]. Previous studies have shown that circRNA is closely related to various ear diseases. For instance, methylation modifications of circRNAs have been implicated in the development and progression of middle ear cholesteatoma [17]. At the molecular level, TMPRSS3 could regulate the viability and apoptosis process of HEI-OC1 cells through circ-Slc4a2 [18]. Moreover, novel_circ_0004013 promoted oxidative stress and apoptosis in HEI-OC1 cells by targeting miR-29a-3p, thereby influencing the progression of ARHL in miR-29a mouse models [19]. Although circRNAs have been implicated in various ear diseases, their expression profiles in serum exosomes from ARHL patients remain unclear. However, circRNA detection is often affected by factors such as tissue specificity and technical limitations, which may lead to low reproducibility of result.

In this study, we employed next generation sequencing to systematically profile the expression of lncRNAs, circRNAs, and mRNAs in serum exosomes of patients with ARHL, and screened out key differentially expressed molecules closely related to the pathogenesis of ARHL. This research is expected to provide new insights into the molecular mechanisms underlying ARHL, providing novel biomarkers and potential therapeutic targets for its early clinical diagnosis and targeted therapy, which has significant theoretical significance and translational value.

## 2. Materials and Methods

### 2.1. Ethical Review and Sample Collection

This study enrolled 6 patients with age-related hearing loss (ARHL) and 6 elderly volunteers with normal hearing as controls. Each patient and volunteer provided written informed consent before sampling. This study was approved by the Medical Ethics Committee of Chongqing General Hospital (approval no. KY S2023-044-01). The clinical information of enrolled participants is summarized in Table 1 and Supplementary Figures S1 and S2.

Participants were divided into two groups: ARHL group and an elderly control group. According to the World Health Organization (WHO) diagnostic criteria for ARHL (WHO, 2021), pure-tone audiometry was conducted in a professional soundproof booth. Participants in the ARHL group were required to have a mean hearing threshold >35 dB HL at four frequencies (0.5, 1, 2, and 4 kHz), while those in the control group were required to maintain thresholds <20 dB HL at the same frequencies.

Inclusion criteria: (1) Over 60 years old. (2) Be capable of completing basic daily living activities, such as dressing, taking a bath, grooming, and using the toilet. (3) The ability to walk independently for 400 m. (4) Right-handed. (5) No previous history of ear trauma or surgery. (6) No other cranial nerve injuries except for cranial nerve VIII. (7) Have not used a hearing aid either currently or before. (8) Sign the informed consent form and be willing to participate in the research.

Exclusion criteria: (1) Noise-induced deafness, Meniere’s disease, herpes zoster oticus, conductive hearing loss, exposure to ototoxic drugs, and other ear diseases with known causes. (2) Cognitive impairment, meningitis, metabolic diseases, cardiovascular diseases, and autoimmune diseases. (3) Previous or current mental or neurological disorders. (4) Addiction to psychoactive substances or sedatives. (5) History of head trauma.

### 2.2. Serum Sampling

Peripheral venous blood was collected and serum was isolated by centrifuging the samples at 1900× *g* for 10 min followed by a second centrifugation at 13,000× *g* for 2 min, both performed at 4 °C. The supernatant was carefully collected after each step. Within each group, three samples were allocated for sequencing and the remaining three for expression validation.

### 2.3. Isolation of Serum Exosomes

After the serum samples were thawed on ice, a series of centrifugation operations were then carried out at 4 °C. Briefly, the samples were first centrifuged at 500× *g* for 5 min to obtain the supernatant. The supernatant was subsequently centrifuged at 2000× *g* for 30 min, and then at 10,000× *g* for 60 min. The resulting supernatant was filtered through a 0.22-μm filter and subjected to ultracentrifugation at 120,000× *g* for 70 min. Discard the supernatant, resuspend the precipitate with sterile PBS to isolate the exosomes for further analysis.

### 2.4. Identification of Serum Exosomes

Nanosight NS300 particle size analyser (NTA): The concentration and size distribution of exosomes were analyzed using a NTA system (NanoSight Technology, Malvern, UK). Before testing, the samples were diluted with sterile PBS to a particle concentration of 10^8^–10^9^ particles/mL and filtered through a 0.22 μm filter. Take 1 mL of the sample and slowly inject it into the sample chamber. Install the laser module and insert the temperature probe. The camera was then activated within the software to adjust the focus and set parameters such as screen gain and camera level. Measurements were performed by selecting the corresponding Standard Operating Procedure (SOP) measurement mode and complete the test.

Transmission electron microscopy (TEM): Exosomes were observed using the JEM-1400 TEM of JEOL Company (JEOL LTD, Peabody, MA, USA). A 20 μL aliquot of the exosome suspension was applied to a copper grid and adsorbed at room temperature for 5 to 10 min. The excess liquid was then removed with filter paper, and the grid was air-dried. After staining with 2% phosphotungstic acid solution for 3 to 5 min, the excess staining solution was aspirated again and dried under an incandescent lamp. Then, electron microscopy observation and image acquisition were carried out.

Western blot: The exosomes were mixed with the sample loading buffer and then placed in a water bath at 100 °C for 5 to 6 min. The membranes were probed overnight at 4 °C with primary antibodies against CD9 (1:1000, Proteintech, Wuhan, China), TSG101 (1:1000, Proteintech), HSP70 (1:1000, Proteintech), and GAPDH (1:10,000, Proteintech). Subsequently, the corresponding secondary antibodies, Goat anti-rabbit IgG (H + L)-HRP (1:5000, Jackson ImmunoResearch, West Grove, PA, USA) and Goat anti-mouse IgG (H + L)-HRP (1:10,000, Jackson ImmunoResearch), were incubated at 37 °C for 1 h, respectively. After washing three times with 1× PBST for 5 min each time, the blots were developed using a chemiluminescent substrate.

### 2.5. Exosomal Transcriptome Sequencing

Total RNA was extracted from exosome samples using RNA extract (Takara, Beijing, China) and chloroform (Takara), followed by precipitation with isopropanol alcohol ((Takara) and washing with 75% ethanol (Sangon, Shanghai, China). Subsequently, micro amplification for 16 cycles was carried out in accordance with the instructions of the RNALib Single cell WTA Kit (Beijing Baiaolaibo Technology Co., Ltd., Beijing China). The resulting cDNA was purified with magnetic beads and quantified using the QuantiFluor dsDNA System (Promega, Madison, WI, USA). Using this cDNA as a template, the sequencing Library was constructed with the Lifeint Transpose DNA Library Prep Kit for Illumina (San Diego, CA, USA), which involved 14 amplification cycles followed by bead-based purification. Finally, the final libraries were quantified with the QuantiFluor dsDNA System (Promega); fragment size was assessed using a Qsep100 S2 cartridge; and PE150 sequencing was carried out on an Illumina NovaSeq 6000 platform (Illumina, San Diego, CA, USA).

### 2.6. Identification of DE-lnRNAs, circRNAs, mRNAsand Functional Analyses

Fastp software (https://github.com/OpenGene/fastp, accessed on 23 March 2025) was used to remove low-quality sequences of the raw reads, including reads with linker sequences, with >10% N bases, all A bases, low-quality bases (Q ≤ 20), or a length < 20 nt. The high-quality clean reads generated were then used for subsequent analysis.

mRNA analysis: Hisat2 software (version 2.2.1-5) was used to compare and analyze the sequencing data with the reference genome. The protein coding gene was quantified and annotated using featureCounts (version 1.0.0.0). The thresholds for significance screening are *p* value < 0.05 and logFC ≥ 1.

lncRNA analysis: Transcript assemblies were generated from the alignment files using Stringtie software (version 2.2.3). These assemblies were then compared against the human reference genome annotation files using gffcompare to identify both known and novel lncRNA transcripts. Transcripts longer than 200 nt were selected as candidate lncRNAs, and their coding potential was predicted using four software programs: CPC2 (version 2.0), CNCI (version 2.0), Pfam (version 37.4), and FEElnc (version 1.0). Transcripts predicted to be coding by at least three tools were filtered out, resulting in a high-confidence set of novel lncRNAs. All lncRNAs (known and novel) were quantified and annotated using featureCounts. The thresholds for significant difference screening are *p* value < 0.05 and logFC ≥ 1.

Cis-target gene prediction for lncRNAs: The functional roles of lncRNAs were explored by predicting cis-regulated target genes, which are protein-coding genes adjacent to their genomic loci. Take the differentially encoded genes lying within 100 kb upstream and downstream of significantly different lncRNAs as their target genes. Subsequently, the main functions of lncRNAs will be predicted through functional enrichment analysis of target genes.

circRNA analysis: circRNAs were identified and annotated using CIRIquant software (https://github.com/bioinfo-biols/CIRIquant, accessed on 24 March 2025), generating a comprehensive annotation file. CircRNA quantification generates two readings based on back spliced junction (bsj) and forward spliced junction (fsj). The software outputted BSJ read counts and the junction ratio (calculated as 2 × BSJ/(2 × BSJ + FSJ)), which were subsequently used for differential expression analysis.

### 2.7. RT-qPCR

Total RNA was extracted from exosomal samples using RNA extract (Takara) according to the manufacturer’s instructions. The extracted RNA was then reverse-transcribed into cDNA using the reverse transcription kit (AU341, TransGen Biotech, Beijing, China). Subsequently, PCR amplification was performed using PerfectStart^®^ Green qPCR SuperMix (AQ601-02, TransGen Biotech), with GAPDH employed as the internal reference gene. The relative expression levels were calculated by the 2^−ΔΔCt^ method. The primer sequence was shown in Table 2.

### 2.8. Statistical Analysis

The plotting Software was Graphpad prism 5 (Graphpad Software, San Diego, CA, USA). *p* < 0.05 and *p* < 0.01 were the screening criteria for significant and extremely significant differences.

## 3. Results

### 3.1. Identification of Serum-Derived Exosomes

**Figure 1:**
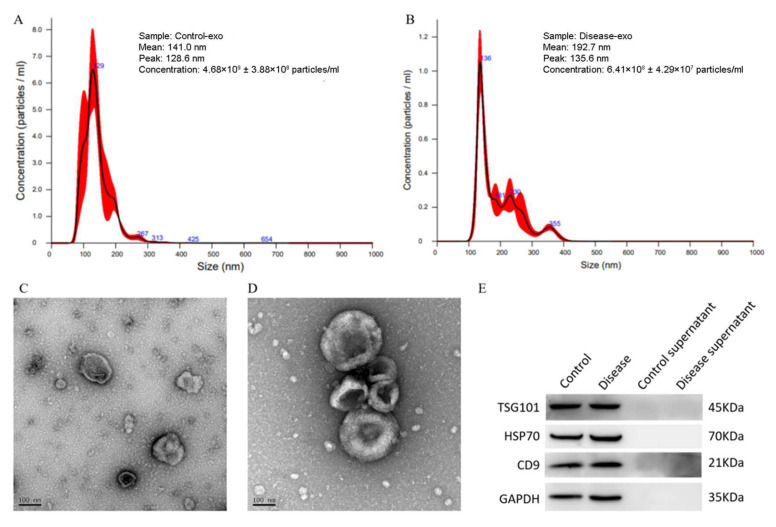
Identification of exosomes isolated from serum of control and ARHL individuals. (**A**,**B**) Particle distribution and average diameter of exosomes measured by NTA. (**C**,**D**) TEM revealed disk-shaped or spherical exosomes with a double-membrane structure. (**E**) Western blot showed TSG101, HSP70, and CD9 were all expressed in exosomes. Control-exo, exosomes isolated from control individuals; Disease-exo, exosomes isolated from ARHL patients; Control-supernatant, supernatant from control individuals; Disease-supernatant, supernatant from ARHL patients.

NTA revealed that the primary peak and average particle size of the majority of exosomes were below 200 nm (Figure 1A,B). TEM demonstrated that exosomes possess a bilayer membrane and typically exhibit spherical or disk-shaped morphologies (Figure 1C,D). Western blot analysis revealed that the exosomal marker proteins TSG101, HSP70, and CD9 were detected in the exosomal fractions, but absent in the corresponding supernatant (Figure 1E). These results demonstrate the successful isolation of exosomes from the serum of both elderly controls and patients.

### 3.2. Quality Control of Sequencing

**Figure 2:**
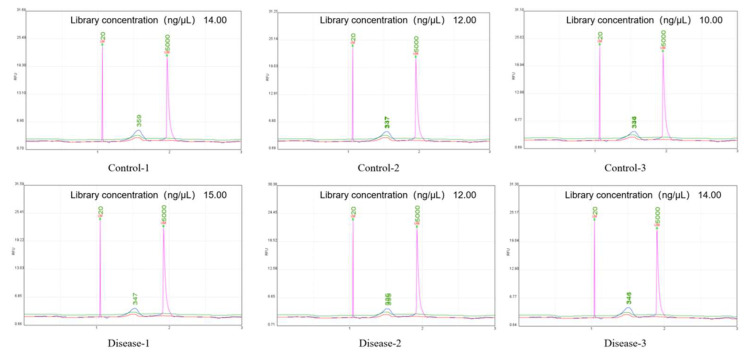
The results of exosomal cDNA Qsep100. The horizontal axis represents the length of cDNA, and the vertical axis represents the fluorescence intensity of the cDNA sample. The purple line represents the signal peak of the library fragment to be tested, while the other different color lines are usually the signal peaks of the “size standard (Marker)” that comes with the instrument, which is used as a ruler for calibrating fragment size.

Library construction was performed with a total cDNA volume ≥ 1 ng (Table 3). The cDNA samples prepared for exosomal sequencing met the required specifications of concentration (≥1 ng/μL) and total quantity (≥500 ng). The Qsep100 detection results indicate that the constructed library has no special circumstances such as cell contamination and meets the sequencing requirements (Figure 2).

### 3.3. Quality Control of Sequence Reads

**Figure 3:**
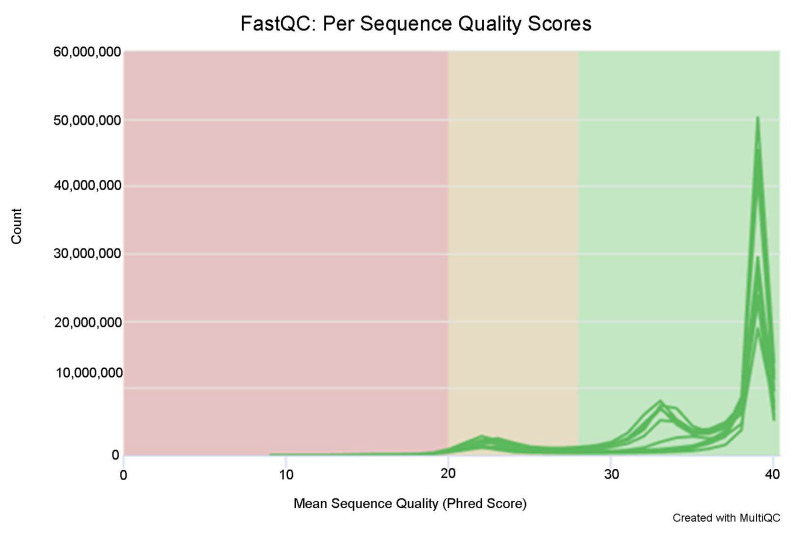
The quality of sequencing bases.

The quality assessment of sequencing output data showed that the clean reads were all above 50 million (Table 4), and the overall quality of sequencing bases met Q30 (Figure 3).

### 3.4. Screening of DE-lncRNAs and Cis-Target Analysis

**Figure 4:**
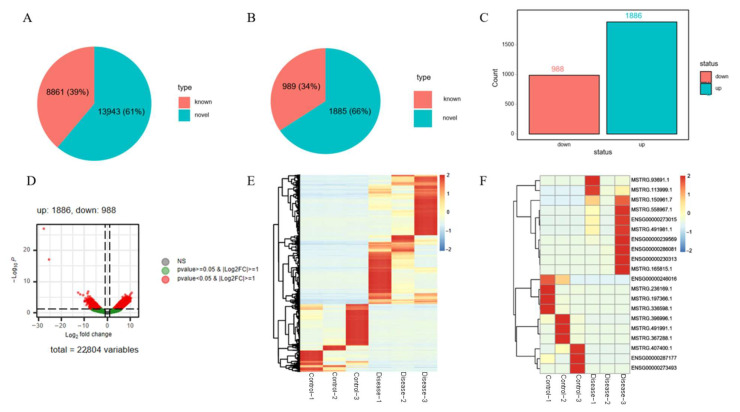
Screening of DE-lncRNAs. (**A**) The expression diagram of lncRNAs. (**B**) The expression diagram of DE-lncRNAs. (**C**) The expression bar chart of DE-lncRNAs. (**D**) The expression volcano chart of DE-lncRNAs. (**E**) The expression heatmap of DE-lncRNAs. (**F**) The expression heatmap of top 20 DE-lncRNAs.

A total of 8861 known lncRNAs and 13,943 novel lncRNAs were identified in the two groups (Figure 4A), among which 2874 were DE-lncRNAs, including 989 known and 1885 novel ones (Figure 4B). The bar chart (Figure 4C) and volcano chart (Figure 4D) showed that 988 DE-lncRNAs were downregulated and 1886 were upregulated. The heatmap demonstrated that the identified DE-lncRNAs distinctly distinguished age-related hearing loss samples from control samples (Figure 4E), with the 20 most significant differences (Figure 4F). A total of 880 cis-target genes were identified for DE-lncRNAs. Through homology comparison analysis of the Ensembl database, we identified homologous or highly similar lncRNAs in mice for the following DE-lncRNAs: MIR223HG, SNHG15, LINCO1952, ROCR, FAM111A-DT, and KMT2E-AS1(Table 5).

### 3.5. Screening of DE-mRNAs

**Figure 5:**
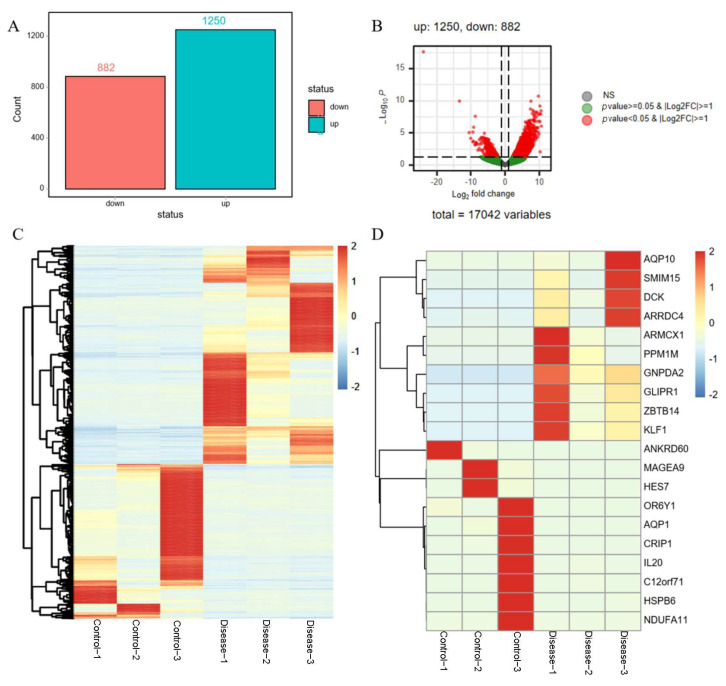
Screening of DE-mRNAs. (**A**) The expression bar chart of DE-mRNAs. (**B**) The expression volcano chart of DE-mRNAs. (**C**) The expression heatmap of DE-mRNAs. (**D**) The expression heatmap of top 20 DE-mRNAs.

A total of 2132 DE-mRNAs were identified (Figure 5A). The bar plot and volcano plot illustrated that 882 DE-mRNAs were downregulated, while 1250 were upregulated (Figure 5B). The heat map shows that the identified DE-mRNAs significantly distinguish ARHL from the control samples (Figure 5C), with the top 20 most significant differences highlighted (Figure 5D).

### 3.6. Screening of DE-circRNAs

**Figure 6:**
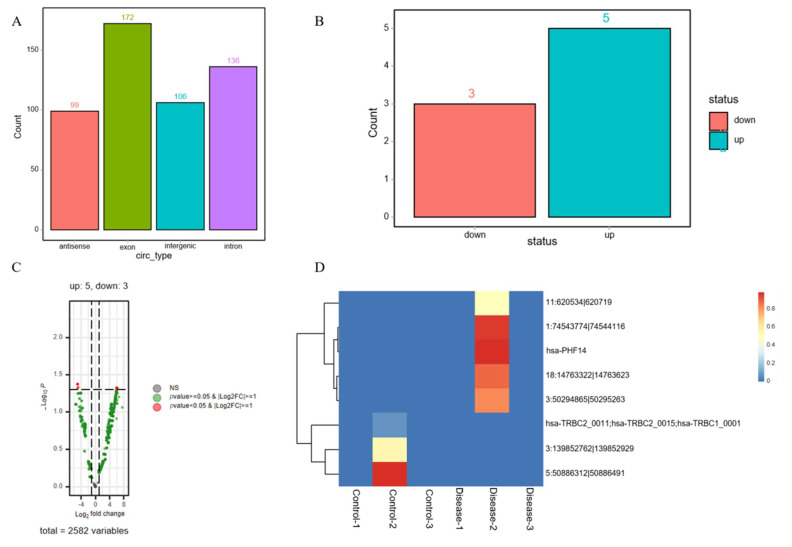
Screening of DE-circRNAs. (**A**) The source bar chart of DE-circRNAs. (**B**) The expression bar chart of DE-circRNAs. (**C**) The expression volcano chart of DE-circRNAs. (**D**) The expression heatmap of DE-circRNAs. Low reproducibility was observed as only one sample per group showed detectable DE-circRNA expression.

A total of 513 known circRNAs were identified, originating from antisense (99), intergenic (106), exon (172), and intron (136) regions (Figure 6A). The bar chart (Figure 6B) and volcano chart (Figure 6C) showed that a total of eight DE-circRNAs were identified in the two groups, with five being upregulated and three downregulated. Notably, heat map analysis showed that only one sample in each group was detected with DE-circRNAs (Figure 6D), suggesting poor reproducibility of these circRNA differential expression results. Therefore, the biological significance of the identified eight DE-circRNAs needs to be further verified in larger sample sizes and independent cohorts.

### 3.7. Chromosomal Distribution

**Figure 7:**
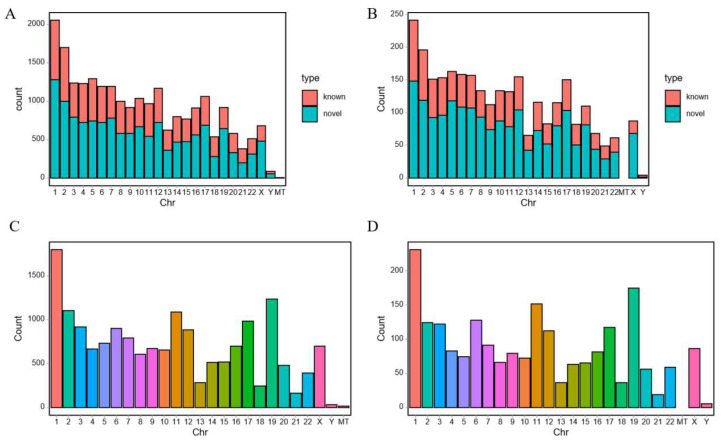
Chromosomal Distribution. (**A**) The bar chart of identified lncRNAs. (**B**) The bar chart of DE-lcRNAs. (**C**) The bar chart of total mRNAs. (**D**) The bar chart of DE-mRNAs.

The chromosomal distribution map revealed that the largest numbers of identified lncRNAs and DE-lcRNAs were located on autosomes 1 and 2 (Figure 7A,B). Similarly, autosomes 1 and 19 harbored the highest numbers of both total mRNAs and DE-mRNAs (Figure 6D and Figure 7C). In contrast, for the sex chromosomes, the quantity of all these RNA species on the X chromosome significantly exceeded that on the Y chromosome.

### 3.8. Enrichment Analysis of Cis-Target Genes and DE-mRNAs

The predicted cis-target genes and DE-mRNAs were, respectively, subjected to enrichment analyses including GO, KEGG, Reactome, and DO.

**Figure 8:**
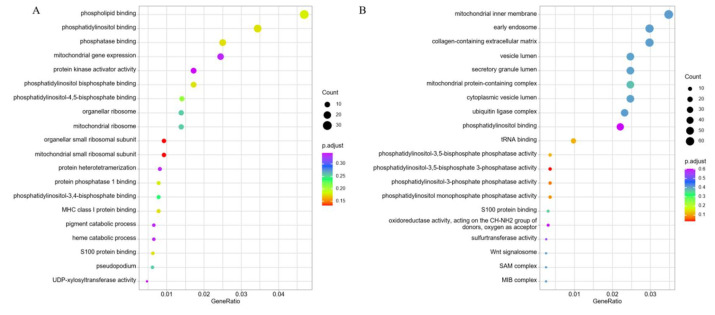
GO functional analyses of predicted cis-target genes and DE-mRNAs. (**A**) Top20 significantly GO terms enriched by the predicted cis-target genes of DE-lncRNAs. (**B**) Top20 significantly GO terms enriched by the DE-mRNAs.

The GO functional annotations of cis-target genes revealed significant enrichment in several critical biological processes and cellular functions, including “phospholipid binding”, “phosphatidylinositol binding”, “phosphatase binding”, “mitochondrial gene expression”, “protein kinase activator activity”, “phosphatidylinositol bisphosphate binding”, “phosphatidylinositol-4,5-bisphosphate binding”, “mitochondrial ribosome”, “organellar small ribosomal subunit”, and “protein phosphatase 1 binding”(Figure 8A). The DE-mRNAs revealed significant enrichment in “mitochondrial inner membrane”, “early endosome”, “collagen-containing extracellular matrix”, “vesicle lumen”, “secretory granule lumen”, “mitochondrial protein-containing complex”, “cytoplasmic vesicle lumen”, “ubiquitin ligase complex”, “phosphatidylinositol binding”, “tRNA binding”, and “phosphatidylinositol-3,5-bisphosphate phosphatase activity”(Figure 8B).

**Figure 9:**
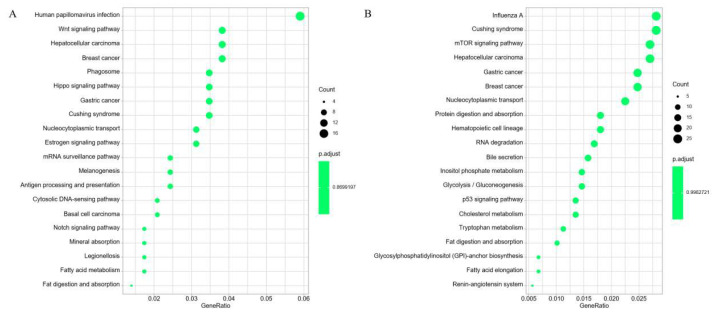
KEGG functional analyses of predicted cis-target genes and DE-mRNAs. (**A**) Top20 significantly KEGG pathways enriched by the predicted cis-target genes of DE-lncRNAs. (**B**) Top20 significantly KEGG pathways enriched by the DE-mRNAs.

The KEGG functional annotation for the cis-target genes was significantly enriched in pathways such as “Human papillomavirus infection”, “Wnt signaling pathway”, “Phagosome”, “Hippo signaling pathway”, “Cushing syndrome”, “Nucleocytoplasmic transport”, “Estrogen signaling pathway”, “mRNA surveillance pathway”, “Antigen processing and presentation”, “Cytosolic DNA-sensing pathway”, and “Fatty acid metabolism”(Figure 9A). The DE-mRNAs revealed significant enrichment in pathways related to “mTOR signaling pathway”, “Nucleocytoplasmic transport”, “Protein digestion and absorption”, “RNA degradation”, “Bile secretion”, “Inositol phosphate metabolism”, “Glycolysis/Gluconeogenesis”, and “p53 signaling pathway”(Figure 9B).

**Figure 10:**
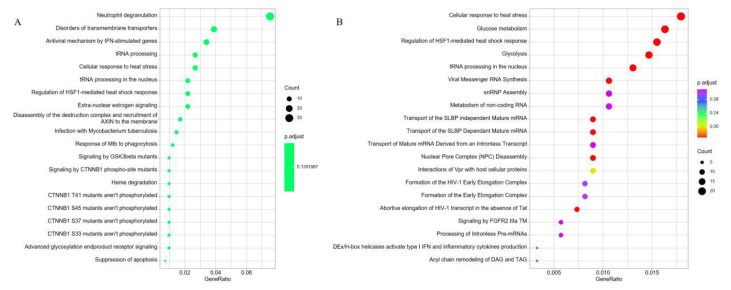
KEGG functional analyses of predicted cis-target genes and DE-mRNAs. (**A**) Top20 significantly Reactome pathways enriched by the predicted cis-target genes of DE-lncRNAs. (**B**) Top20 significantly Reactome pathways enriched by the DE-mRNAs.

Reactome pathway analysis for the cis-target genes were associated with “Neutrophil degranulation”, “Disorders of transmembrane transporters”, “Antiviral mechanism by IFN-stimulated genes”, “tRNA processing”, “Cellular response to heat stress”, “Extra-nuclear estrogen signaling”, and “Suppression of apoptosis” (Figure 10A). The DE-mRNAs were predominantly enriched in pathways related to “Cellular response to heat stress”, “Glucose metabolism”, “Regulation of HSF1-mediated heat shock response”, “Glycolysis”, “tRNA processing in the nucleus”, “snRNP Assembly”, “Metabolism of non-coding RNA”, “Transport of the SLBP independent Mature mRNA”, “Signaling by FGFR2 IIIa TM”, and “Acyl chain remodeling of DAG and TAG” (Figure 10B).

**Figure 11:**
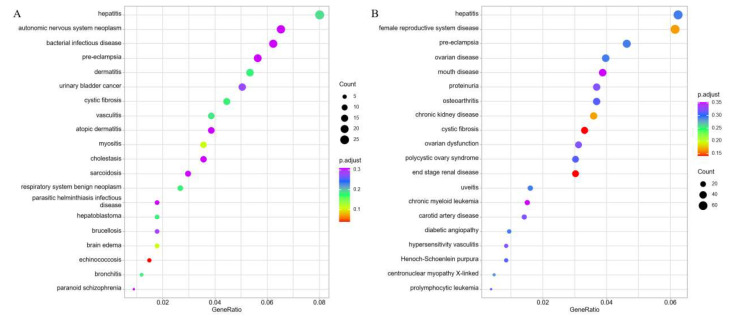
DO functional analyses of predicted cis-target genes and DE-mRNAs. (**A**) Top20 significantly DO pathways enriched by the predicted cis-target genes of DE-lncRNAs. (**B**) Top20 significantly DO pathways enriched by the DE-mRNAs.

The DO functional annotation of cis-target genes were significantly enriched in non-specific enrichment terms such as “autonomic nervous system neoplasm”, “hepatitis”, “bacterial infectious disease”, “pre-eclampsia”, “cystic fibrosis”, “vasculitis”, “brain edema”, “paranoid schizophrenia”, “myositis”, “cholestasis”, and “sarcoidosis” (Figure 11A). The DE-mRNAs revealed significant enrichment in non-specific enrichment terms including “hepatitis”, “diabetic angiopathy”, “female reproductive system disease”, “pre-eclampsia”, “ovarian disease”, “osteoarthritis”, “cystic fibrosis”, “carotid artery disease”, “hypersensitivity vasculitis”, “centronuclear myopathy X-linked”, and “prolymphocytic leukemia” (Figure 11B).

### 3.9. Verification of Sequencing by RT-qPCR

**Figure 12:**
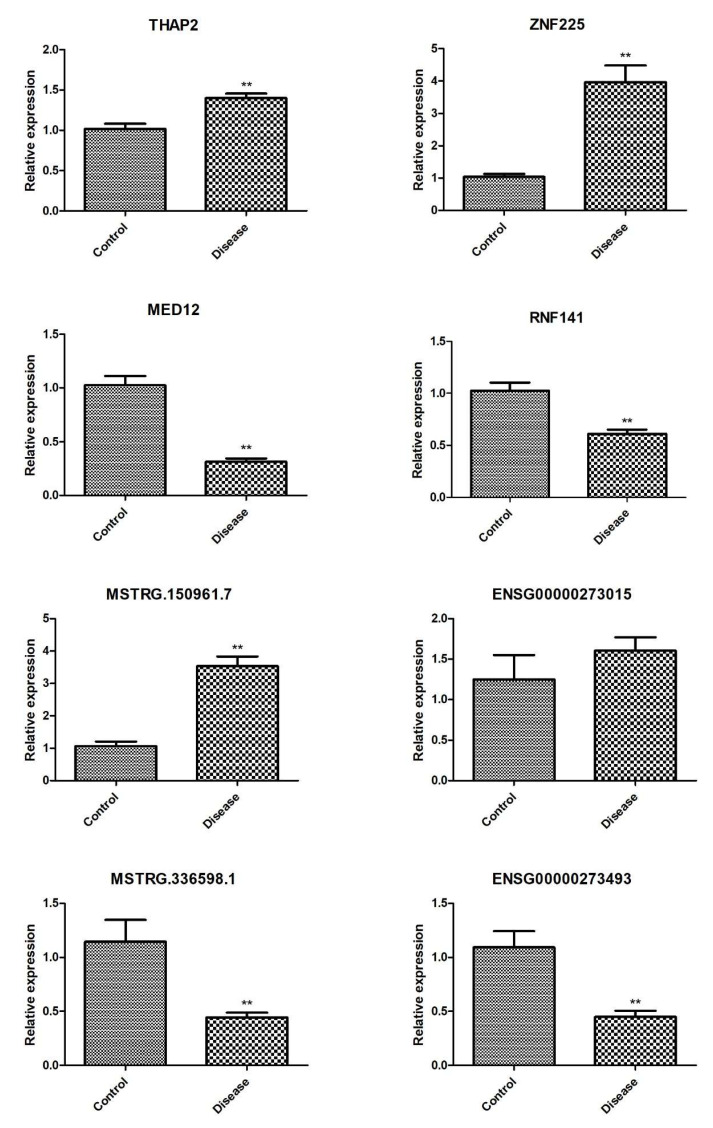
Expression analysis of exosomal mRNAs (*THAP2*, *ZNF225*, *MED12*, *RNF141*) and lncRNAs (MSTRG.150961.7, MSTRG.336598.1, ENSG00000273493, ENSG00000273015) (n = 3). ** *p* < 0.01 vs. controls.

RT-qPCR analysis revealed the expression levels of *THAP2*, *ZNF225*, and MSTRG.150961.7 were all highly significantly upregulated, Conversely, the expression levels of *MED12*, *RNF141*, MSTRG.336598.1, and ENSG00000273493 were downregulated in the ARHL group, while the expression of ENSG00000273015 showed no significant difference (Figure 12).

## 4. Discussion

The pathogenesis of ARHL is influenced by multiple factors. Currently, its clinical diagnosis and therapeutic effect evaluation still mainly rely on a comprehensive assessment scheme combining clinical symptoms and audiological examinations, which presents challenges such as difficulties in early diagnosis and limited treatment options. Therefore, exploring the key regulatory factors and potential therapeutic targets for age-related hearing loss has become a critical challenge that needs to be urgently addressed in current clinical practice.

Med12 plays a critical role in the development of many tissues. Its mutations are associated with X-linked intellectual disability syndrome and hearing loss [20,21], while the gene also regulates adult auditory function by maintaining the structural integrity of the stria vascularis [22]. Our sequencing analysis revealed a significant downregulation of *MED12* expression in the serum of patients with ARHL. This result was further verified by RT-qPCR, suggesting that MED12 may play an important role in the pathogenesis of ARHL.

As widely expressed in the inner ear and involved in secretion and absorption processes [23,24], AQP1 has been confirmed to be closely associated with hearing loss [25]. It has shown expression changes in various middle ear diseases, such as cholesteatoma otitis media, exudative otitis media related to bacterial infection, and Zika virus infection models [26,27]. A pioneering study in 2024 first suggested a potential link between AQP1 and ARHL. The study found that in the cochlear sensory epithelium of C57BL/6J mice, the expression of AQP1 showed an upregulated trend with increasing age [28]. Based on this, our study further reveals a significant downregulation of AQP1 levels in the serum of patients with ARHL, providing a new perspective for deep exploration of its underlying mechanisms.

LncRNA SNHG15 is an important regulatory factor for many diseases. Studies have demonstrated that it promotes gastric cancer progression by interacting with the HNRNPA1/SLC7A11/GPX4 pathway to regulate ferroptosis [29]; stabilizes nucleolin to drive pre-osteoblast proliferation [30]; facilitates host cell invasion of SARS-CoV-2 via modulating RABL2A [31]; and augments the tumorigenicity in myeloma by increasing chromatin accessibility and recruiting the histone modifier SETD2 to promote H3K36me3 modification [32] and modulates hypoxic–ischemic brain injury via the miR-153-3p/SETD7 axis [33]. Although its roles in various diseases have been gradually revealed, a direct association with hearing loss remains unexplored. Here, we report for the first time the significant upregulation of lncRNA SNHG15 in serum exosomes of patients with ARHL, implicating its important role in the disease process and highlighting the need for further research.

Functional enrichment analyses of DE-lncRNA cis-target genes and DE-mRNAs identified multiple pathways, but only those with clear biological relevance to ARHL are discussed herein, while non-specific signals are explained and de-emphasized. GO analysis showed that both cis-target genes and DE-mRNAs are significantly enriched in terms such as “phosphatidylinositol binding” and “phosphatidylinositol-4,5-bisphosphate binding”. Previous studies have shown that phosphatase and phosphatidylinositol phosphatase are involved in the regulation of hair cell survival [34] and play an important role in maintaining hearing [35]. For example, phosphatidylinositol 4,5-bisphosphate is essential for the activity of the KCNQ4 potassium channel in hair cells [36], and its abnormal upregulation can lead to ciliary dysfunction in hair cells, thereby causing progressive hearing loss [37]. Aminoglycosides can not only inhibit the KCNQ4 channel in cochlear outer hair cells by consuming phosphatidylinositol 4,5-bisphosphate [38], and also bind to phosphatidylinositol diphosphate to inhibit its hydrolysis and disrupt the integrity and structure of the membrane, which leads to changes in the non-specific permeability of the membrane and causes ototoxicity [39]. This suggested that DE-lncRNAs and DE-mRNAs may regulate ARHL by modulating phosphatidylinositol-mediated ion channel function. However, further experimental research and functional validation of its specific regulatory mechanisms are warranted.

For the Wnt/β-catenin signaling pathway, it is essential for the development of the auditory system. Its enhanced activity facilitates the differentiation of human pluripotent stem cells into hair cell-like cells [40] and promotes the mitotic regeneration of cochlear hair cells [41]. Conversely, ethylbenzene can inhibit this pathway, leading to mitochondrial damage and excessive apoptosis in cochlear progenitor cells, thereby leading to hearing loss [42]. However, the activity of this pathway is downregulated during aging. For instance, in a D-galactose-induced aging rat model, the Wnt/β-catenin signaling in the auditory cortex is weakened, and activation of the pathway by lithium chloride alleviates apoptosis and neurodegeneration in the auditory cortex [43]. In this study, through the analysis of cis-target genes, it was suggested that lncRNAs may be involved in the regulation of ARHL through the Wnt signaling pathway, but there are currently few related studies.

Reactome analysis showed significant enrichment of cellular response to heat stress in both cis-target genes and DE-mRNAs. Heat stress can increase the amount of f-actin in the outer hair cells of the cochlea in mice and make them harder [44]. Preconditioning with heat stress not only protects outer hair cells from traumatic noise damage by enhancing their rigidity but also improves distortion product otoacoustic emissions [45]. In rats, adaptation to low levels of stress (30 days at 34 degrees C) activates cellular mechanisms that elevate antioxidant levels, better tolerate subsequent different types of severe stress, and prevent permanent noise-induced hearing loss [46]. However, there are relatively few related studies on the relationship between heat stress and ARHL. Taken together, we speculate that dysregulation of heat stress response may contribute to ARHL pathogenesis and progression.

DO functional enrichment analysis suggests that inflammation plays a significant role in the pathogenesis of ARHL. Cohort studies have found that the inflammatory marker C-reactive protein is significantly elevated in individuals with ARHL [47]. In animal models, an increase in macrophages in the cochlea of aged CBA/CaJ mice and activation of microglia in the cochlear nucleus were observed [48]. In the cochlea of aged C57BL/6J mice, the expressions of inflammation-related genes TLR4, Myd88, NF-κB, and IL-1β were significantly upregulated. Long-term caffeine supplementation has been shown to ameliorate ARHL by downregulating the TLR4/NF-κB inflammatory pathway [49]. Notably, aging is often accompanied by a chronic, low-grade inflammatory state in tissues and organs, which can lead to various diseases [50,51]. For instance, pathogens and metabolites of intestinal microorganisms can be transported to the cochlea through the systemic circulation, causing chronic cochlear inflammation and resulting in ARHL [52]. These pieces of evidence collectively support that inflammation is a key pathogenic factor in ARHL.

However, terms such as “hepatitis”, “bacterial infectious disease” (DO analysis), and “human papillomavirus infection” (KEGG analysis) are considered non-specific. These may arise from the inclusion of irrelevant cis-target genes, heterogeneous cell sources of exosomal RNAs, or statistical false positives. They have no known association with auditory system function or ARHL pathogenesis and are not discussed further.

In addition, regarding circRNAs, although we identified eight DE-circRNAs, the low reproducibility across samples indicates that these results should be interpreted with extreme caution. The potential reasons include the high tissue-specificity and individual variability of circRNA expression, technical challenges in detecting low-abundance circRNAs in serum exosomes, and possible subtype heterogeneity of ARHL. For complexity of circRNA biogenesis and tissue specificity, circRNAs are generated by back-splicing of pre-mRNAs, and their formation is regulated by multiple factors such as splicing regulators, RNA-binding proteins, and genomic sequences [53,54]. The low reproducibility may be attributed to the high tissue-specificity and individual variability of circRNA expression, which is consistent with previous studies reporting that circRNA expression patterns are more heterogeneous than mRNAs and lncRNAs in clinical samples [55,56]. For technical limitations in circRNA detection, although we used CIRIquant software (a widely recognized tool for circRNA identification and quantification) and strictly controlled the sequencing quality (Q30 > 90%), the detection of circRNAs in serum exosomes may be affected by factors such as low abundance of circRNAs, potential contamination during exosome isolation, and biases in library construction. These technical challenges could lead to inconsistent detection of circRNAs across samples. For potential ARHL subtype heterogeneity, the low reproducibility may also reflect the existence of unknown subtypes of ARHL with distinct circRNA expression profiles. ARHL is a complex multifactorial disease, and individual differences in genetic background, environmental exposures, and disease progression may lead to heterogeneous circRNA expression patterns [57]. Given the current limitations, the identified DE-circRNAs cannot be considered as reliable candidate molecules for ARHL, and further studies with larger sample sizes, optimized detection techniques, and subtype stratification are required to clarify the role of circRNAs in ARHL pathogenesis.

## 5. Deficiencies

Although we have identified a series of novel DE-lncRNAs and DE-circRNAs, the specific functions of these lncRNAs in ARHL remain largely unexplored. The scarcity of existing literature on this topic underscores the exploratory nature of our study. Furthermore, the expression inconsistency exhibited by DE-circRNAs suggests the possible existence of unknown ARHL subtypes or the complex pathophysiological mechanisms inherent in ARHL. In addition, technical factors such as low abundance of circRNAs in serum exosomes and potential biases in library construction may also contribute to the low reproducibility. Therefore, the biological significance of the identified DE-circRNAs urgently needs to be further clarified in subsequent work with larger sample sizes and improved detection methods. In addition to the unclear functions of novel DE-lncRNAs and low reproducibility of circRNA results, the other two key limitations also should be acknowledged. First, the qPCR validation with three samples per group is underpowered, limiting the statistical reliability of the experimental verification. Second, the overall cohort size (six samples per group) is modest, which may affect the generalizability of the findings. Therefore, subsequent work will focus on expanding the sample size to 20–30 samples per group in independent cohorts to validate the expression of candidate mRNAs/lncRNAs, assess their diagnostic potential, and perform functional experiments to clarify their roles in ARHL pathogenesis.

## 6. Conclusions

This study provides the first systematic profile of lncRNA and mRNA expression in serum exosomes from ARHL patients, identifying 2874 DE-lncRNAs and 2132 DE-mRNAs. Functional enrichment analyses revealed potential involvement of phosphatidylinositol signaling, Wnt signaling, cellular response to heat stress, and inflammatory pathways in ARHL pathogenesis. Preliminary qPCR validation (n = 3 per group) showed consistent expression trends for four key mRNAs (*THAP2*, *ZNF225*, *MED12*, and *RNF141*) and three DE-lncRNAs (MSTRG.150961.7, MSTRG.336598.1, ENSG00000273493), suggesting these molecules may serve as potential candidate biomarkers or pathogenic regulators. However, due to the small sample size (six per group for the main study, three per group for qPCR validation), the current data are exploratory in nature. Future studies with larger independent cohorts (20–30 samples per group) are urgently needed to validate the expression stability of these candidate molecules, confirm their clinical utility as biomarkers, and explore their specific functional mechanisms in ARHL.

## Figures and Tables

**Table 1:** The general condition and hearing information of the subjects.

Group	No	Sex	Age	Pure Tone Hearing, dB HL (Right)						Pure Tone Hearing, dB HL (Left)						Aim
				250 Hz	500 Hz	1000 Hz	2000 Hz	4000 Hz	8000 Hz	250 Hz	500 Hz	1000 Hz	2000 Hz	4000 Hz	8000 Hz	
ARHL	1	Male	60	40	45	55	45	50	60	40	45	55	50	55	70	Sequencing
ARHL	2	Male	66	45	40	45	45	55	70	40	45	45	50	60	65	
ARHL	3	Female	65	35	35	40	45	50	65	35	35	45	45	55	65	
ARHL	4	Male	76	50	45	30	25	75	65	60	55	30	30	80	70	RT-qPCR
ARHL	5	Female	74	50	50	55	55	60	75	50	50	55	60	75	70	
ARHL	6	Female	76	70	80	80	80	75	80	50	60	60	65	60	95	
Control	1	Female	80	10	5	10	10	20	20	5	10	15	10	20	25	Sequencing
Control	2	Male	62	10	10	10	15	25	25	5	15	10	15	25	20	
Control	3	Male	62	5	5	20	20	20	25	5	10	20	20	25	25	
Control	4	Female	78	30	25	20	25	30	45	35	30	20	20	35	40	RT-qPCR
Control	5	Female	73	25	20	25	20	25	35	20	20	20	15	20	30	
Control	6	Male	68	20	20	10	5	10	10	20	20	10	5	10	5	

**Table 2:** Details of PCR primers utilized in this investigation.

Name of Primer	Primer Sequence (5′-3′)
THAP2-F	GACCTAACAGGACAAACTCGAC
THAP2-R	TTCCTAAAGGCATAGCTGTGTTC
ZNF225-F	GAGGAAACTGTACCGAGAAGTG
ZNF225-R	CAACCTGGCAGGGCATATCAT
MED12-F	GGGATCTTGAGCTACGAACAC
MED12-R	GCAGGCTGGTTATTGAAACCTTG
RNF141-F	AGGTACAACCTGGGTCTGATT
RNF141-R	GATCCGTGATGCCTCCACAAT
MSTRG.336598.1-F	CTTTCCCAAGCCTAAACTCC
MSTRG.336598.1-R	CCAGGTCCCTCCTACAACAC
ENSG00000273493-F	GTGAGCCTCTGTACTCAGCC
ENSG00000273493-R	AGCATCCGTATAATCCCAAC
MSTRG.150961.7-F	GGCAAATAGAATACAGGCAAAG
MSTRG.150961.7-R	CCAAGGAGGAGACAGCACAT
ENSG00000273015-F	TCGGGTAGAGGCACTGGTAT
ENSG00000273015-R	CCTGAACTTTCTGGCTTTGT
GAPDH-F	TGACAACTTTGGTATCGTGGAAGG
GAPDH-R	AGGCAGGGATGATGTTCTGGAGAG

**Table 3:** The cDNA concentration of exosomes constructed by the library.

Sample	cDNA Concentration (ng/μL)	cDNA Amount (ng)
Control-1	0.135	2.025
Control-2	0.268	4.02
Control-3	0.114	1.71
Disease-1	0.502	7.53
Disease-2	1.49	22.35
Disease-3	0.298	4.47

**Table 4:** The clean reads output.

File	reads_raw	Q20_raw	Q30_raw	raw_GC%	reads_clean	Q20_clean	Q30_clean	clean_GC%
Control-1_R1	78,656,385	92.76%	82.71%	55%	73,764,681	97.57%	91.62%	55%
Control-1_R2	78,656,385	95.39%	89.16%	55%	73,764,681	98.06%	93.47%	56%
Control-2_R1	79,677,271	93.14%	87.29%	54%	72,423,083	96.85%	90.55%	53%
Control-2_R2	79,677,271	93.31%	85.71%	54%	72,423,083	97.26%	91.97%	54%
Control-3_R1	95,244,292	91.03%	79.57%	57%	88,743,759	97.16%	90.82%	58%
Control-3_R2	95,244,292	94.55%	87.58%	58%	88,743,759	97.79%	92.83%	59%
Disease-1_R1	52,277,157	92.90%	83.83%	52%	47,240,551	97.56%	92.44%	52%
Disease-1_R2	52,277,157	94.59%	88.54%	52%	47,240,551	97.90%	93.93%	52%
Disease-2_R1	83,257,263	89.39%	76.82%	50%	73,886,370	96.46%	89.94%	47%
Disease-2_R2	83,257,263	92.77%	85.38%	50%	73,886,370	97.13%	92.31%	48%
Disease-3_R1	86,928,320	90.93%	79.64%	54%	79,369,320	97.07%	90.95%	54%
Disease-3_R2	86,928,320	93.79%	86.62%	54%	79,369,320	97.49%	92.48%	54%

**Table 5:** Co-expressed DE-lncRNAs in humans and mice.

Gene ID	Chr	Type	Gene Name	p.adj
1	X	known	MIR223HG	0.00481856657084325
2	7	known	SNHG15	0.0176116108001056
3	7	known	LINC01952	0.0177821566059439
4	17	known	ROCR	0.0191986215485735
5	11	known	FAM111A-DT	0.019429101307526
6	7	known	KMT2E-AS1	0.0211511805849077

## Data Availability

The RNA-seq data generated in this study have been deposited in the NCBI Sequence Read Archive (SRA) database under the BioProject accession number PRJNA1391672. The data are publicly accessible via the following: https://www.ncbi.nlm.nih.gov/bioproject/PRJNA1391672 (accessed on 30 December 2025).

## Supplementary Materials

The following supporting information can be downloaded at: https://www.mdpi.com/article/10.3390/diagnostics16020248/s1, Figure S1: The audiograms of age-related hearing loss (Disease) cases (n = 6); Figure S2: The audiograms of elderly control cases (n = 6).
