# Supplementary material for: The Whole Transcriptome Sequencing Profile of Serum-Derived Exosomes and Potential Pathophysiology of Age-Related Hearing Loss
Source: Diagnostics (Basel). 2026 Jan 12;16(2):248. doi: 10.3390/diagnostics16020248 (PMC12839563; doi:10.3390/diagnostics16020248)
Supplement: Supplementary file 1 [file diagnostics-16-00248-s001.zip › diagnostics-4027651 supplementary materials.pdf]

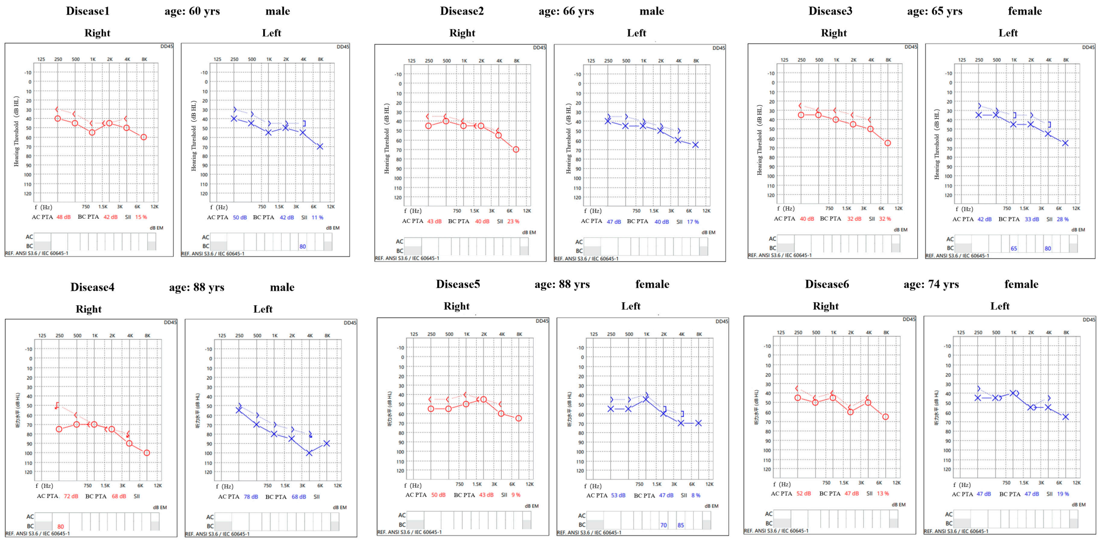

Figure S1. The audiograms of age-related hearing loss (Disease) cases (n=6).

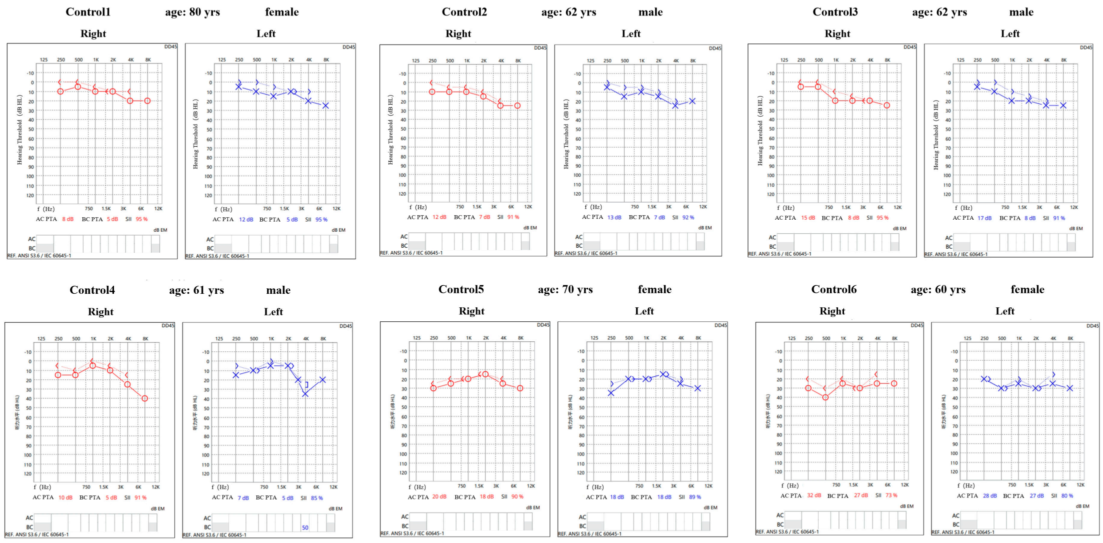

Figure S2. The audiograms of elderly control cases (n=6).
